# Genetics Underlying an Individualized Approach to Adult Spinal Disorders

**DOI:** 10.3389/fsurg.2016.00061

**Published:** 2016-11-22

**Authors:** Corey T. Walker, Phillip A. Bonney, Nikolay L. Martirosyan, Nicholas Theodore

**Affiliations:** ^1^Department of Neurosurgery, St. Joseph’s Hospital and Medical Center, Barrow Neurological Institute, Phoenix, AZ, USA

**Keywords:** cervical spondylotic myelopathy, genetics, genome-wide association study, heritability, intervertebral disc degeneration, ossification of posterior longitudinal ligament, proteomics

## Abstract

Adult spinal disorders are a significant cause of morbidity across the world and carry significant health and economic burdens. Genetic predispositions are increasingly considered for these conditions and are becoming understood. Advances in molecular technologies since the mid-1990s have made possible genetic characterizations of these diseases in many populations, and recent findings have provided insight into the underlying pathophysiologic mechanisms. These studies have made clear the genetic heterogeneity producing clinical phenotypes and suggest that individualized treatments are possible in the future. We review the genetics and heritability of cervical spondylotic myelopathy and ossification of the posterior longitudinal ligament and perform a systematic review of the genetics of adult lumbar degenerative scoliotic deformity, highlighting recent discoveries and the potential for personalized future therapeutics for these patients.

## Introduction

Degenerative diseases of the spine affect the majority of individuals over a lifetime, causing pain and neurologic dysfunction, and presenting a significant challenge to physicians. These clinical entities develop from a complex interplay of genetic and environmental factors that are incompletely understood. Patients with a given disease may appear similar radiographically, yet outcomes may be disparate regarding disease progression and responses to medical, rehabilitative, or surgical interventions. Although there is a role for surgery in certain conditions, patient selection must be carefully considered, given the risks associated with surgery. Better stratification of patients is needed to guide treatments.

Advances in molecular technologies over the last 15 years have provided much insight into the genetics of numerous diseases, including those of the spine. Candidate gene approaches and genome-wide association studies have shed light on underlying pathophysiologic mechanisms at work in the development of such diseases. The potential application of these studies to patient care includes providing a clearer picture of who will benefit from surgical intervention. Furthermore, they offer an exciting opportunity for future therapies targeting specific genetic aberrations that predispose to disease.

Another article in this issue of *Frontiers* highlights the genetics of intervertebral disc (IVD) disease, which may play a partial role in almost all degenerative spine disorders and, therefore, is not discussed here. Alternatively, we review the genetics of several of the most common spinal disorders, including cervical spondylotic myelopathy (CSM), ossification of the posterior longitudinal ligament (OPLL), and adult scoliosis. Rather than enumerate specific single nucleotide polymorphisms (SNPs) and other genetic anomalies, we highlight the overarching mechanisms uncovered by recent studies and discuss the potential for development of personalized approaches to treating these diseases in the future.

## Methods

### Literature Review

We performed a systematic review of the literature to evaluate the contributions of current evidence on the genetics of adult degenerative scoliosis (DS). A recent systematic review that evaluated the genetic contributions for CSM and OPLL by Wilson et al. (1) was found and therefore was not performed in this study. The inclusion criteria included the studies comparing genetic variables in humans with this disease. Only studies in the English language were included. MEDLINE was queried with the terms “genetics” and “adult degenerative scoliosis” for articles published from 1966 to September 26, 2016. Studies focusing on other forms of scoliosis were excluded, as were those focusing solely on IVD degeneration. These queries returned 24 studies. The citation information for each result was examined by two of the authors for relevant studies. Six potentially relevant studies were identified, and the abstracts (and, if necessary, full manuscripts) of these studies were reviewed. The references of all reviewed manuscripts were also reviewed to identify other potential studies. Six studies met the inclusion criteria and were the focus of the present study. The studies were published between 2011 and 2015. All of the studies represented Level III evidence (small, non-randomized case–control studies). Results of the included studies were extracted and interpreted by the two reviewing authors.

## Cervical Spondylotic Myelopathy

Cervical spondylosis is a nearly ubiquitous finding that occurs with aging as IVD degeneration, ligamentous laxity, facet hypertrophy, and osteophyte formation contribute to narrowing of the spinal canal (2). CSM occurs when neural elements of the spinal cord are compressed. Nevertheless, many patients incidentally show radiographic evidence of spinal cord compression but remain clinically asymptomatic (3). Although the exact reasons for this remain unknown, a potential explanation relates to the dynamic nature of the cervical spine and cord, and that static compression does not correlate exactly with the micropathological changes that occur in this disease (4).

Multiple human and animal studies have implicated various mechanisms in the acute and chronic pathophysiology of CSM (5). Direct mechanical forces result in static and dynamic injuries to neuronal and glial cells (6, 7). This injury is likely paralleled by ischemic changes seen in the disease caused by obstructed spinal cord perfusion and consequent microvascular changes (7–10). Several studies have also suggested a perpetuating cycle of ischemia related to blood–spinal cord barrier breakdown and dysregulation of the neurovascular unit (11, 12). Vascular permeability promotes edema through the release of inflammatory molecules and other potentially cytotoxic proteins into the cord parenchyma (13). This edema may potentiate neuronal damage and play an active role in the chronic degenerative component of the disease (14, 15). Additionally, glutamatergic toxicity (16), free radical-mediated cell injury (17, 18), and apoptosis (19) are also suggested as aggravating secondary injury pathways in the disease.

### Heritability of CSM

An appreciation for the genetics of a disease is important for understanding how it is passed from one generation to the next. Although environmental factors undoubtedly play a role in the multifactorial pathogenesis, they allow providers and patients to assess the probability that the patient could develop the disease. Meaningful genealogy, however, is difficult to evaluate, and studies are limited (20). A study by Wilson et al. systematically reviewed the literature documenting the heritability of CSM and OPLL (1). Several authors have suggested genetic susceptibility of cervical spondylosis in twin–twin comparison studies (21–23). However, only one study has successfully used a population-based methodology to show inheritance patterns among non-twins (24). Patel et al. examined a database of 2 million Utah residents and found 486 patients with CSM and compared them with 1000 case controls (24). They used an index measuring genetic distance between pairs of patients to quantify familial clustering and found a statistically significant relationship related to the disease. Moreover, they identified a greater than five times relative risk of developing the disease among first-degree relatives. Studies corroborating these findings in other populations need to be done, but the data suggest heritability among the studied individuals.

### Genetics of CSM

As methods for evaluating genomics have evolved, SNPs and proteomics have become easier to evaluate, and the literature regarding their contributions to CSM has grown. Nevertheless, identifying individual components of CSM is difficult as different genes spur degenerative changes that lead to spondylosis but not necessarily myelopathy. For example, Wang et al. have associated two different genetic polymorphisms with CSM (25). First, they identified two polymorphisms of the vitamin D receptor gene (*VDR*), *Apa*I and *Taq*I, which are related to the presence of CSM and the magnetic resonance imaging–based severity of disease in Chinese patients (25). They also found a strong link between CSM and the tryptophan allele (Trp2) of the collagen 9A2 gene, as well as smoking exposure (26). These polymorphisms promote IVD degeneration independently (27–32), a process that can cause central canal stenosis. Neither of these studies delineated how these genetic changes compared in patients with cervical stenosis with and without myelopathy. This also underlines the putative effects of environmental stressors on pathogenesis.

Another genetic relationship has been drawn between apolipoprotein E, a protein that plays a critical role in the repair and regeneration processes of multiple central nervous system diseases. Specifically, the ε4 allele of the apolipoprotein E gene is implicated in impairment of these repair mechanisms. In a study by Setzer and colleagues, 106 patients with radiographic cervical stenosis were collected prospectively, and the ε4 allele was strongly associated with the development of CSM, independent of imaging findings, and other confounders (the allele was not related to the degree of stenosis) (33). The group showed that the allele also had negative effects on treatment outcomes in 60 of the patients who underwent surgical decompression (34). These results suggest that this genetic link portends a worse prognosis both for developing the disease and recovering from it. Large-scale studies evaluating the clinical usefulness of this association are necessary before its application can become widespread. Still, this knowledge may provide clues to the pathogenesis of the disease and potential therapeutic targets.

## Ossification of the Posterior Longitudinal Ligament

Ossification of the posterior longitudinal ligament is a condition of ectopic bone formation within the posterior longitudinal ligament, typically occurring at the cervical spine levels. It was first described as a disease of aging in Asian populations, with a prevalence of approximately 1–4%, though the prevalence is reported to be as high as 1.7% in Caucasian populations (35). About 17% of individuals with OPLL present with cervical myelopathy, while 29% of asymptomatic OPLL patients go on to develop myelopathy over the next three decades (36). Additionally, OPLL adds complexity to the treatment of cervical spondylosis and, ultimately, affects the surgical approach to treating symptomatic patients (37). Studies of the natural history of OPLL are clouded by the common presence of other coexisting degenerative spinal pathologies.

Little is known about the exact pathophysiologic mechanisms underlying OPLL. Multiple factors are suspected to play roles in the ectopic bone formation, including numerous biomechanically and metabolically mediated growth factors and cytokines (38). *In vivo* findings from human OPLL samples demonstrate degenerative elastic and cartilaginous fibers with metaplastic, hypertrophic cartilage cells (38, 39). Neovascularization, vascular endothelial growth factor-positive metaplastic chondrocytes, and abnormal collagen expression are thought to play a role in the spreading endochondral ossification front (38, 39). Additional studies are required to expand our understanding of OPLL and likely will be influenced by the wealth of genomic and proteomic results.

### Heritability of OPLL

Several genetic studies have been performed to establish the heritability of OPLL. A study of 347 families of patients with OPLL found a 26% prevalence in parents and a 28% prevalence in siblings (40). In this study, the relative risk of first-degree relatives developing the disease was statistically significant and greater than five times that of the expected incidence in the general population. Another study looking at approximately 100 patients and relatives with OPLL found a prevalence of 27% with a relative risk of seven times that of the general population (41). Although a high segregation rate among siblings and a high prevalence of disease in parents suggest an autosomal dominant pattern of inheritance, neither study showed autosomal dominant (or recessive) inheritance on further analysis. Likewise, a polygene inheritance hypothesis was also rejected in these studies (40, 41). Altogether, these data suggest a high rate of heritability but not in a predictable fashion that would allow for practical genetic counseling.

### Genetics of OPLL

Multiple genes have been targeted as possible contributors to the pathogenesis of OPLL. One of the first to be investigated was the ectonucleotide pyrophosphatase/phosphodiesterase (*ENPP1*) gene, a transmembrane metalloenzyme that regulates soft-tissue calcification and bone mineralization *via* the production of inorganic pyrophosphate, a known inhibitor of calcification (42). *ENPP1* was first implicated after studies in *ttw* mice showed altered gene expression causing tiptoe walking (43). The mice harbor a naturally recessive mutation that results in ectopic spinal ligament ossification and myelopathy that mirrors the disease traits in human OPLL (43). Several case–control studies in humans have examined SNPs in the *ENPP1* gene, the main enzyme that controls inorganic pyrophosphate in osteoblasts and chondrocytes. The results have linked various polymorphisms to disease susceptibility, severity, and location, but the results have been inconsistent regarding which SNPs are involved (44–46). Nonetheless, these findings implicate *ENPP1* as a possible therapeutic target. Further work needs to be done to elucidate the exact mechanisms by which it is modified in OPLL.

Collagen molecules have also received significant attention in the genetic research for OPLL. Mutations in type XI collagen within the *COL11A2* gene are thought to affect the formation of fibril networks in the extracellular matrix and change the conformation of Type II collagen, which is responsible for bone and cartilage formation (38). Two large genome linkage studies found five different SNPs in *COL11A2* that correlated with disease presence, and one was present in both reports (47, 48). Type VI collagen is also associated with multiple SNPs in chromosome 21, localizing to the *COL6A1* gene (49). Other studies have shown similar findings and linked this SNP to ossification of the ligamentum flavum and diffuse idiopathic skeletal hyperostosis (50, 51). However, a study of these *COL6A1* SNPs in a Korean population revealed conflicting results (52). Although significant data support the two collagen molecules as contributing to the pathogenesis of OPLL, lack of data congruency has made reliable conclusions difficult to make, likely due to disease heterogeneity.

Bone morphogenetic proteins and transforming growth factor-β have been studied extensively due to their role in physiological and pathological pathways of bone formation and metabolism. Several SNPs are associated with both of these proteins, specifically bone morphogenetic protein-2, bone morphogenetic protein-4, and transforming growth factor-β1 (53–58). Although fewer studies have focused on these molecules and replication studies still are needed, they present attractive targets for future research. Likewise, multiple other candidate genes have been investigated independently (Table 1) (43–51, 53–66). A full list of SNP associations is well summarized in other studies (1, 67, 68).

**Table 1:** **Genetics of ossification of the posterior longitudinal ligament**.

Gene	Reference
Collagen VI	Tanaka et al. (49), Tsukahara et al. (51), Kong et al. (50)
Collagen XI	Koga et al. (59), Maeda et al. (48), Maeda et al. (60), Sakou et al. (47)
RXRβ	Numasawa et al. (61)
Vitamin D receptor	Shiigi et al. (62), Kobashi et al. (53)
ENPP1	Okawa et al. (43), Nakamura et al. (45), Koshizuka et al. (44), Tahara et al. (46)
BMRF	Ogata et al. (54)
CTGF/Hcs24	Yamamoto et al. (55)
BMP-2	Kawaguchi et al. (56), Tanaka et al. (57), Kawaguchi et al. (58), Wang et al. (63)
TGFβ	Kawaguchi et al. (56), Kamiya et al. (64), Horikoshi et al. (65)
Osteopontin	Aiba et al. (66)

*RXRβ, retinoic X receptor-β; ENPP1, ectonucleotide pyrophosphatase/phosphodiesterase 1; BMRF, bone metabolism regulatory factor; CTGF, connective tissue growth factor; Hcs24, hypertrophic chondrocyte-specific gene product 24; BMP, bone morphogenetic protein; TGFβ, transforming growth factor β*.

The first genome-wide association study for OPLL identified 26 SNPs on 3 chromosomes at 8p11.21, 8q23.1, 8q23.3, 12p11.22, 12p12.2, and 20p12.3 that are considered to be significantly associated with OPLL. Six of those SNPs were confirmed in a replication test as highly susceptible gene loci for OPLL (69). Interestingly, their comparison with previously reported gene loci from prior studies uncovered no significant associations. Two of the genes, radial spoke head 9 homolog, *RSPH9* (coding for a protein that composes cilia and plays a role in the hedgehog pathway of skeletal development), and serine/threonine kinase 38 like, *STK38L* (a protein kinase that inhibits cell cycle progression), are believed to have a part in the pathobiology of OPLL through membranous ossification (69). Hydroxyacid oxidase 1, *HAO1* (encodes hydroxyacid oxidase 1, which oxidizes 2-hydroxyacid), R-spondin 2, *RSPO2* (encodes R-spondin 2 protein that contributes to osteoblastogenesis through Wnt/β-catenin signaling pathways), and coiled-coil domain containing 91, *CCDC91* (encodes a trans-Golgi network protein) have putative roles in the endochondral ossification process (69). A follow-up study by the same group focused on *RSPO2* and further implicated it by evaluating the putative SNP *in vitro* and how it affected the binding of a vital transcription factor, CCAAT-enhancer-binding protein β (C/EBPβ) (70). Macroscopically, these associations remain speculative at this time; nevertheless, the cumulative findings of these studies open the door for investigation of multiple new gene targets and provide insight into the novel mechanisms of OPLL.

## Degenerative Lumbar Scoliosis

DS is a disease that occurs after skeletal maturity, typically after the third decade of life, and is a distinct entity from idiopathic scoliosis. It is associated with severe back and leg pain, which leads to spinal dysfunction and debilitation. Pain may result from asymmetric muscular loading, facet joint arthritis, or nerve root impingement/traction (71). A Cobb angle of greater than 10° in the coronal plane is considered diagnostic (72). Although it has been recognized for many decades as a significant cause of pain and disability, increasing clinical awareness has yielded a growing body of literature on treatment and greatly improved clinical outcomes.

Although interest in outcomes and surgical treatments has gained ground, little is known about the pathogenesis of DS. Multiple studies have implicated osteoporosis in DS. There is a high degree of overlap of these two pathologies; however, a causal relationship has not been established, and definitive correlations are lacking (73). Other studies suggest that asymmetric IVD degeneration is the cause, resulting in uneven loading forces that perpetuate the rate of asymmetric degeneration. Recent evidence suggests that cytokines and growth factors are differentially expressed within various locations of the IVD, likely creating regional discrepancies in the rates of cellular apoptosis, inflammation, and angiogenesis (74–76). Whether or not these differences are the result of other causative etiologies or are the instigating impetus behind the disease remains to be seen. Investigations into other sources of asymmetric spinal degeneration, such as myopathy and mechanical instability, are lacking and provide future directions for research (76, 77).

### Heritability of DS

Few studies have been performed to examine the heritability of DS to date. Twin–twin and family-based comparison studies should be conducted to help identify patterns of inheritance. The paucity of data likely is due to the relatively recent attention to this disease since the mid-1990s. Unlike OPLL, which is found at particularly high rates in specific parts of the world, DS appears to show less geographic variation.

### Genetics of DS

For adolescent idiopathic scoliosis (AIS), a number of investigations have been conducted to examine the genetic basis of disease. In 2010, Ward et al. performed a genome-wide association study that identified 53 SNPs that correlated with scoliotic curvature in Caucasian females (78). Using this genotype information and an initial Cobb angle, they devised an algorithm that calculated the risk of curvature progression in selected patients, which they commercialized under the name *ScoliScore*. This DNA-based predictive calculator theoretically enabled clinicians to forecast which individual patients were at low likelihood of curve progression (78). However, replication studies failed to demonstrate the same SNP associations in different geographically diverse populations (79–82). Nevertheless, this attempt at personalized, genome-based, clinical and outcome prediction exemplifies methods by which genetic outcomes could guide future treatments for a multitude of diseases.

Comparatively, in DS, there have been fewer genetic incongruities identified than in other degenerative spine diseases. It is thought that distinct genetic characteristics define these seemingly similar, but quite clinically different syndromes. Our systematic review identified six works in the literature (Table 2) (52, 83–87) that identified genetic contributions for DS. The quality of the data is relatively limited (Level III studies) but provides some insight into potential genetic mechanisms for disease pathogenesis that could potentially be used in future studies.

**Table 2:** **Review of studies identifying genetic contributions to adult degenerative scoliosis**.

Reference	Level of evidence	Genetic level of participation	Putative contributor(s)
Zhu et al. (83)	III	Proteomics	CLU, Ficolin-3
Han et al. (84)	III	Proteomics	PIAS2, NDUFA2, TRIM68
Shin et al. (85)	III	Copy number variation	TMEM163, ANKRD 11, NFATC1
Hwang et al. (86)	III	SNPs	rs2276454 of collagen type II alpha 1
Kim et al. (87)	III	SNPs	No SNPs of NMDA receptor genes associated
Kim et al. (52)	III	SNPs	RIMS2

*CLU, clusterin; PIAS2, protein inhibitor of activated STAT 2; NDUFA2, NADH:ubiquinone oxidoreductase subunit A2; TRIM68, tripartite motif containing 68; TMEM163, transmembrane protein 163; ANKRD 11, ankyrin repeat domain 11; NFATC1, nuclear factor of activated T-cells, cytoplasmic, calcineurin-dependent 1; SNPs, single nucleotide polymorphisms; NMDA, N-methyl-d-aspartate; RIMS2, regulating synaptic membrane exocytosis 2*.

Proteomic analyses of the sera of patients with DS have identified 11 proteins that are differentially expressed in such patients, 2 of which were secondarily confirmed with Western blot analyses, CLU (also known as apolipoprotein J, testosterone-repressed prostate message-2, SP 40-40, complement lysis inhibitor, gp80, glycoprotein III, and sulfate glycoprotein-2) and Ficolin-3 (also named Hakata antigen, thermolabile β-2 macroglycoprotein, thermolabile substance, and H-ficolin) (83). Both of these proteins have been suggested to have roles in autoimmunity, although they both likely have multiple roles in the human body and their association is non-specific. This protein expression analysis does not definitively implicate them in the disease pathogenesis but suggests that they may serve as potential future biomarkers and potentially raises the question of whether an autoimmune component of DS exists. The same group also compared proteomic expression in cultured mesenchymal stem cells of DS patients (84). This comparison revealed differential levels of three proteins, protein inhibitor of activated STAT 2 (PIAS2), NADH:ubiquinone oxidoreductase subunit A2 (NDUFA2), and tripartite motif containing 68 (TRIM68), none of which correspond to those elevated in the serum. All three of these proteins play various roles in biological processes and, therefore, pinpointing their roles may be difficult. Although the above proteomic analysis is helpful in identifying biomarkers of disease and drug targets, the proteins characterized in the analysis do not correlate exactly with genetic differences in the disease and are susceptible to environmental and other outside factors that change the genomic output *via* epigenetic influences. Consequently, much more work is required to tease out the meaning of these proteomic differences and reproduce these results with different patient populations.

One of the studies comparing genomic differences in DS examined copy number variations, which represent regional gene dosages of DNA segments 1 kb or larger (85). Of the 260 copy number variations identified by microarray analysis, quantitative polymerase chain reaction validation identified three genes with significant differences from the control group. These genes included transmembrane protein 163, *TMEM163*, a gene coding for a transmembrane protein of unknown function; ankyrin repeat domain 11, *ANKRD11*, an ankyrin repeating gene implicated in autism spectrum disorder and skeletal formation; and nuclear factor of activated T cells, cytoplasmic, calcineurin-dependent 1, *NFATC1*, a gene reportedly involved in bone mineral density (85). This novel study provided evidence that DS could be predisposed by inherent genomic differences rather than resulting from external environmental forces causing asymmetric degeneration. Further work will be needed to expand on the biosignaling cascades by which these genes may affect disease pathogenesis.

Likewise, another area of gene-based research relates to SNPs associated with DS. Prior studies have given collagen molecules significant attention for similar diseases, including IVD degeneration and AIS (88). Collagen II has been investigated because of its structural role in stress-bearing of the spine (88). Investigators studied SNPs of *COL2A1* and found a significant association of SNP (rs2276454) in *COL2A1* to DS in Korean patients (86). Another study tested SNPs of glutamate receptors (*N*-methyl-d-aspartate receptors), given their role in controlling bone remodeling through stimulation, maturation, and differentiation of osteoblasts and osteoclasts (87). Interestingly, they found no association with any of the SNPs investigated. A similar study found that one of the SNPs in regulating synaptic membrane exocytosis 2, *RIMS2*, coding for a presynaptic active zone protein that regulates vesicle exocytosis of neurotransmitters, was significantly associated with DS (87). Therefore, glutamate, or other neurotransmitters, may still contribute to the disease progression of DS. The heterogeneity of these findings suggests that our insight into DS remains minimal; yet, these results lay the groundwork for further basic science in this area.

## Conclusion

Adult degenerative spinal disease has tremendous health costs on a global level. CSM, OPLL, and DS have gained significant attention from medical providers and researchers as disease entities that merit further focus and investigation (Table 3). Studies of families and twins suggest that there may be a significant component of heritability to CSM and OPLL, although few genetic studies of DS have been published. Thus, DS presents an opportunity for further research. Advances in genomic analysis and biostatistics continue to open new doors to finding genetic linkages with these degenerative spinal diseases, which ultimately could guide the course of future pathophysiological studies, clinical diagnoses, and treatments.

**Table 3:** **Summary of cervical spondylotic myelopathy (CSM), ossification of the posterior longitudinal ligament (OPLL), and lumbar degenerative scoliosis (DS)**.

CSM
Complex disease with multiple degenerative processes contributing to underlying spondylosis
Studies support an inherited predisposition to the disease
Several genes have been implicated in CSM, but more studies are needed to confirm their genetic role in the disease
OPLL
Data support the heritability of OPLL, and first-degree family members are at a much higher risk than others
*Col6A1* and *Col11A2* are suggested by multiple studies to be associated with OPLL
Multiple SNPs have been implicated in OPLL, along with several new genes from a genome-wide association study, but more work is needed to confirm their involvement
DS
No studies have established any inherited predisposition to DS
Fewer studies examining genetic associations have been performed for DS compared to CSM and OPLL, but there appear to be genetic contributions to DS
More studies are required to identify participating genetic alterations in the disease pathogenesis

## Author Contributions

All the authors made substantial contributions to the conception or design of the work.

## Conflict of Interest Statement

The authors declare that the research was conducted in the absence of any commercial or financial relationships that could be construed as a potential conflict of interest.

## Abbreviations

AIS, adolescent idiopathic scoliosis; CSM, cervical spondylotic myelopathy; DS, degenerative scoliosis; IVD, intervertebral disc; OPLL, ossification of posterior longitudinal ligament; SNP, single nucleotide polymorphism.
